# A new nomogram of urinary flow rate and volume based on multiple measurements per healthy adult Japanese men using a portable uroflowmeter (P-Flowdiary®)

**DOI:** 10.1186/s12894-022-01086-5

**Published:** 2022-08-25

**Authors:** Masatake Shinohara, Kazumasa Torimoto, Chie Matsushita, Daisuke Gotoh, Hisashi Yoshida, Toshihisa Saka, Yoshihiko Hirao, Akihide Hirayama, Kiyohide Fujimoto

**Affiliations:** 1 Department of Urology, Osaka Gyoumeikan Hospital, Osaka, Japan; 2 Department of Urology, Nara Medical University, Kashihara, Japan; 3 Department of Urology, Saiseikai Chuwa Hospital, Sakurai, Japan; 4 Faculty of Biology-Oriented Science and Technology, Kindai University, Kinokawa, Japan; 5 Department of Urology, Kindai University Nara Hospital, Ikoma, Japan

**Keywords:** Age, Man, Nomogram, Urinary flow rate, Urinary volume

## Abstract

**Background:**

To develop a nomogram of urinary volume and flow based on the data of Japanese men without lower urinary tract symptoms and multiple flows per participant whose characteristics were clear.

**Methods:**

Overall, 101 Japanese male volunteers without lower urinary tract symptoms aged between 20 and 59 years were enrolled. A portable uroflowmeter (P-Flowdiary®) was used to record urinary information (flow rate and volume) for 2 successive days. The model (quadratic, linear, or logarithmic regression) most fit for the relationship between maximum flow rate and voided volume was determined. The maximum flow rate at > 150 mL was compared among the 20–29-, 30–39-, 40–49-, and 50–59-year age groups. Nomograms appropriate for the age groups were created.

**Results:**

The mean age, International Prostate Symptom Score, and Overactive Bladder Symptom Score were 38.5 years, 0.42, and 0.24, respectively. The quadratic regression model was the most fit because its mean coefficient determination was 0.93 ± 0.06. The mean maximum flow rate was significantly lower in the 50–59-year age group (21.8 ± 5.05 mL/s, *P* < 0.01) than in the younger groups (24.14 ± 4.94, 24.05 ± 6.99, and 24.64 ± 5.72 mL/s). The 2 nomograms are *Y* = 28.99 {1 − exp(− 0.01 × *X*)} and *Y* = 25.67 {1 − exp(− 0.01 × *X*)} for the 20–49- and 50–59-year age groups, respectively.

**Conclusions:**

The nomogram can predict maximum flow rate based on voided volume in Japanese men aged 20–59 years without lower urinary tract symptoms.

## Introduction

The basic noninvasive evaluation tools for voiding dysfunction are lower urinary tract symptom (LUTS) questionnaires, such as the International Prostate Symptom Score (IPSS), Overactive Bladder Symptom Score (OABSS), and Core LUTS score [1–3], uroflowmetry (UFM) with measurement of postvoid residual volume, and frequency volume chart (FVC) [4, 5]. UFM and FVC, as objective tools, provide real information on the voiding pattern, whereas LUTS questionnaires, as participantive tools, may show information different from that of real voiding. Usually, UFM is only performed once per patient in the clinics due to time restriction. However, such routine UFM does not always reproduce typical voiding because in the clinics, patients often do not have full bladder enough to void or cannot void well due to the different environment compared to private toilets. To resolve these problems, UFM should be performed several times per patient with FVC ideally at home, or nomograms should be used that accurately predict multiple urinary flow based on voided volume (VV) and flow rate at onetime micturition.

**Fig. 1:**
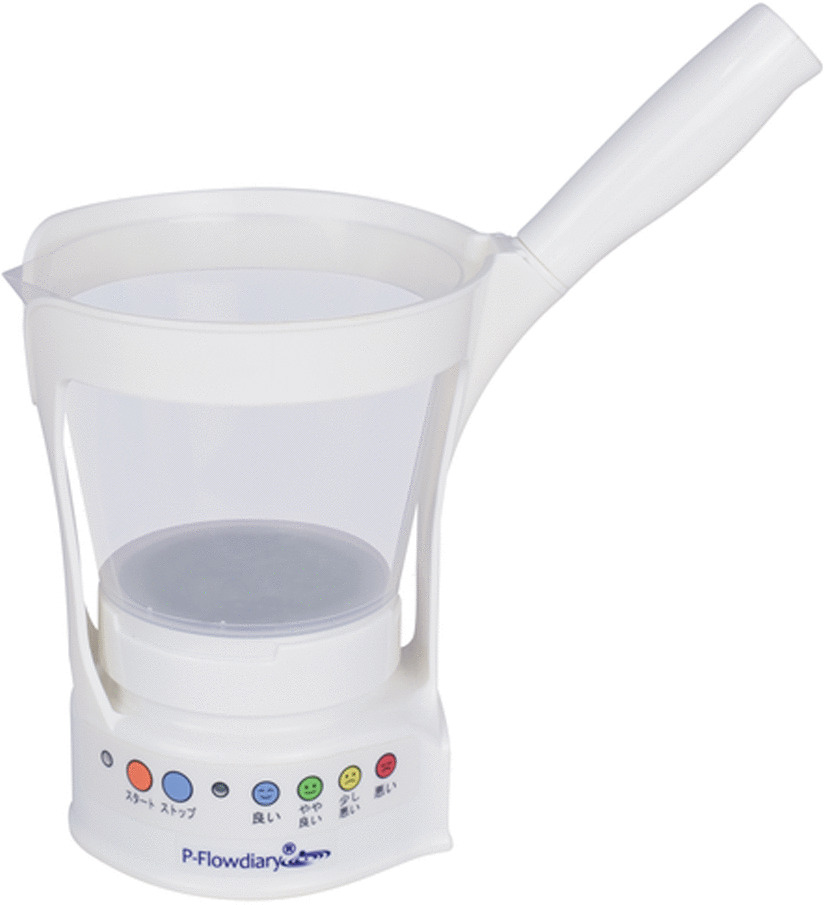
P-Flowdiary®

A portable (home-use) uroflowmeter, which electrically and automatically records flow trace and VV per micturition, is useful for measuring multiple usual micturitions [6–9]. We developed a portable uroflowmeter, P-Flowdiary® (Muranaka Medical Instruments Co., Ltd., Osaka, Japan), which has a disposable plastic cup, as urine reservoir and automatically records date, time, flow rate, and volume with simple switch operation (Fig. 1).

Nomograms, which show the relationship between VV and flow rates, were developed previously [10–12], but these nomograms have limitations. The first nomogram was made in Canada in 1973 [13] and was assessed in 1979 [10]. Those data were based on 300 flows of 80 men without LUTS, and the mean number of flows per participant was 3.8. However, the ages of the participants were unknown. The Liverpool nomogram was made in England, and it was based on the data of 331 men without LUTS and 1 flow per participant [12]. The mean age of the participants was 49 years (range, 16–64 years). In the study, the maximum flow rates (MFRs) decreased with age (1.0–1.6 mL/s/10 years of age). Therefore, 2 nomograms were made: one for men younger than 50 years and another for men 50 years or older. However, the details of the participants’ characteristics were unknown. A Japanese nomogram was made based on the data of 233 flows in 13 men (age range, 26–45 years) without LUTS [11]. The study did not consider the influence of age. To make a useful tool to assist in diagnosing voiding dysfunction, we aimed to develop a new nomogram of urinary volume and flow based on the data of a sufficient number of Japanese men without LUTS and multiple flows per participant whose characteristics were clear.

## Methods

Japanese male volunteers without LUTS with ages between 20 and 59 years were enrolled from September 2015 to May 2019. The IPSS and OABSS were used to assess LUTS. Volunteers who had a composite score of IPSS ≥ 6 and urgency score of OABSS ≥ 2 were excluded. The P-Flowdiary® was used to record urinary information, including the date, time, flow rate, and volume, for 2 successive days. P-Flowdiary® is a small and lightweight medical device approved for use in Japan. The device measures urinary volume in the cup with a gravimetric sensor on the cup holder. Weight is set to zero once the device is turned on, and the volume and flow rate are measured based on weight changes. All recorded data were stored in the SD card inside the device and retrieved as Excel files. The participants were educated using the device manual and were able to learn how to use it in < 30 min. Artifacts appeared as steep spikes on the curves if the cup of the device was touched and could be visually distinguished. Urinary curves with such artifacts were excluded from the analysis.

The following steps were performed:

- Step 1: The relationship between VV and MFR was examined based on multiple data sets on urinary flows of each participant. We ascertained the model which was most fit among the following: quadratic, linear, or logarithmic regression.
- Step 2: Previous studies suggest that VV > 150 mL is necessary for an accurate evaluation of flow rates [14, 15]. Therefore, we compared the MFR between the age groups using VV data of ≥ 150 mL. We evaluated whether the relationship between VV and MFR was different between the age groups (20–29, 30–39, 40–49, and 50–59 years).
- Step 3: Nomograms on VV and MFR were created by age group.

All data were expressed as mean ± standard deviation. Ordinary 1-way analysis of variance and Dunnett’s multiple comparison test were used to analyze statistical differences. A *P*-value < 0.05 was defined as statistically significant.

This study was performed in accordance with the ethical principles of the Declaration of Helsinki. The protocol was approved by the Ethics Committee of Osaka Gyoumeikan Hospital (Approval number: 17–0023). All participants provided written informed consent before enrollment.

## Results

A total of 101 participants were enrolled, and their characteristics are presented in Table 1. All the participants had no lower urinary symptoms and no history of urological disease. If any, the participants were not registered. The mean IPSS and OABSS were less than 1.0, which indicated that the probability issues related to lower urinary tract function were extremely low.

**Table 1:** Background of participants

Age groups (years)	Number of participants	Frequency	Age (years), mean ± SD	Weight (kg), mean ± SD	BMI (kg/m^2^), mean ± SD	Maximum flow rate (mL/s), mean ± SD	Mean flow rate (mL/s), mean ± SD	Voided volume (mL), mean ± SD	IPSS, mean ± SD	QOL, mean ± SD	OABSS, mean ± SD
20–29	30	282	24.8 ± 2.5	62.9 ± 10.4	21.5 ± 2.8	20.5 ± 6.7	7.5 ± 3.6	203.9 ± 115.3	0.2 ± 0.4	0.1 ± 0.3	0.1 ± 0.3
30–39	25	183	34.5 ± 2.8	66.4 ± 10.8	22.6 ± 3.4	22.1 ± 7.6	7.9 ± 3.1	241.4 ± 122.2	0.28 ± 0.72	0.08 ± 0.27	0.16 ± 0.46
40–49	21	180	43.4 ± 2.9	70.9 ± 10.1	24.3 ± 3.6	22.9 ± 7.5	8.7 ± 3.8	285.4 ± 142.8	0.48 ± 0.79	0.14 ± 0.35	0.33 ± 0.64
50–59	25	249	55.0 ± 2.9	67.8 ± 11.2	22.4 ± 3.3	18.9 ± 6.6	7.5 ± 3.5	228.3 ± 135.4	0.8 ± 1.2	0.24 ± 0.65	0.44 ± 0.64
Total	101	894	38.5 ± 11.8	67.5 ± 11.8	22.8 ± 3.5	21.3 ± 6.9	8.1 ± 3.4	244.1 ± 129.1	0.42 ± 0.72	0.13 ± 0.31	0.24 ± 0.5

IPSS, International Prostate Symptom Score; QOL, Quality of Life score; OABSS, Overactive Bladder Symptom Score; SD, standard deviation

### Step 1

**Fig. 2:**
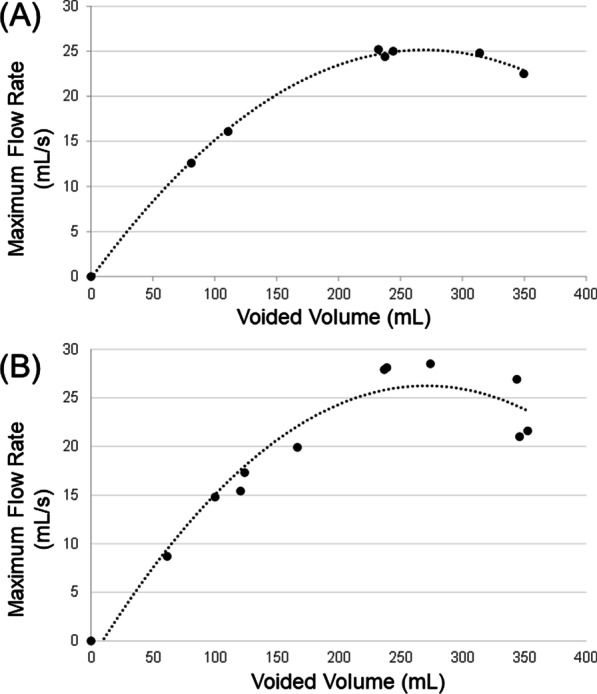
Representative graphs of the quadratic regression models: **A** 28-year-old man (R^2^ = 0.9488) and **B** 52-year-old man (R^2^ = 0.9269)

The quadratic regression model was significantly fitter than the linear and logarithmic regression models (Table 2). Two representative graphs of the quadratic regression model were shown, using the data of a 28- and a 52-year-old man (Fig. 2A and B).

**Table 2:** Comparison of the coefficients of determination (R^2^) among the 3 regression models to represent the relationships between voided volume and maximum flow rate

Age groups (years)	Coefficient of determination (R^2^), mean ± SD		
	Quadratic regression	Linear regression	Logarithmic regression
20–29	0.92 ± 0.06	0.70 ± 0.17****	0.82 ± 0.13**
30–39	0.93 ± 0.06	0.65 ± 0.21****	0.81 ± 0.11**
40–49	0.94 ± 0.05	0.74 ± 0.17****	0.85 ± 0.09*
50–59	0.91 ± 0.07	0.66 ± 0.21****	0.83 ± 0.10
20–59	0.93 ± 0.06	0.69 ± 0.21****	0.83 ± 0.13****

Data are described as mean ± standard deviation

Ordinary 1-way analysis of variance and Dunnett’s multiple comparison test were used

^*^P < 0.05, **P < 0.01, ***P < 0.001, ****P < 0.0001: compared with quadratic regression

### Step 2

The mean MFR was significantly lower in the 50–59-year age group than in the younger groups. The mean MFRs were not significantly different among the 20–29-, 30–39-, and 40–49-year age groups (Table 3). Therefore, we decided to make 2 nomograms, one for the younger groups (20–29, 30–39, and 40–49) and another for the 50–59-year age group.

**Table 3:** Maximum flow rates with a voided volume > 150 mL

Age group (years)	Frequency	Maximum flow rate (mL/s), mean ± SD	95% Confidence interval
20–29	179	24.14 ± 4.94***	23.41–24.87
30–39	143	24.05 ± 6.99**	22.90–25.21
40–49	158	24.64 ± 5.72****	23.74–25.54
50–59	177	21.8 ± 5.05	21.05–22.55
Total	657	23.61 ± 5.76	23.17–24.05

Data are described as mean ± standard deviation

Ordinary 1-way analysis of variance and Dunnett’s multiple comparison test were used

^**^P < 0.01, ***P < 0.001, ****P < 0.0001: compared with the 50–59-year age group

### Step 3

**Fig. 3:**
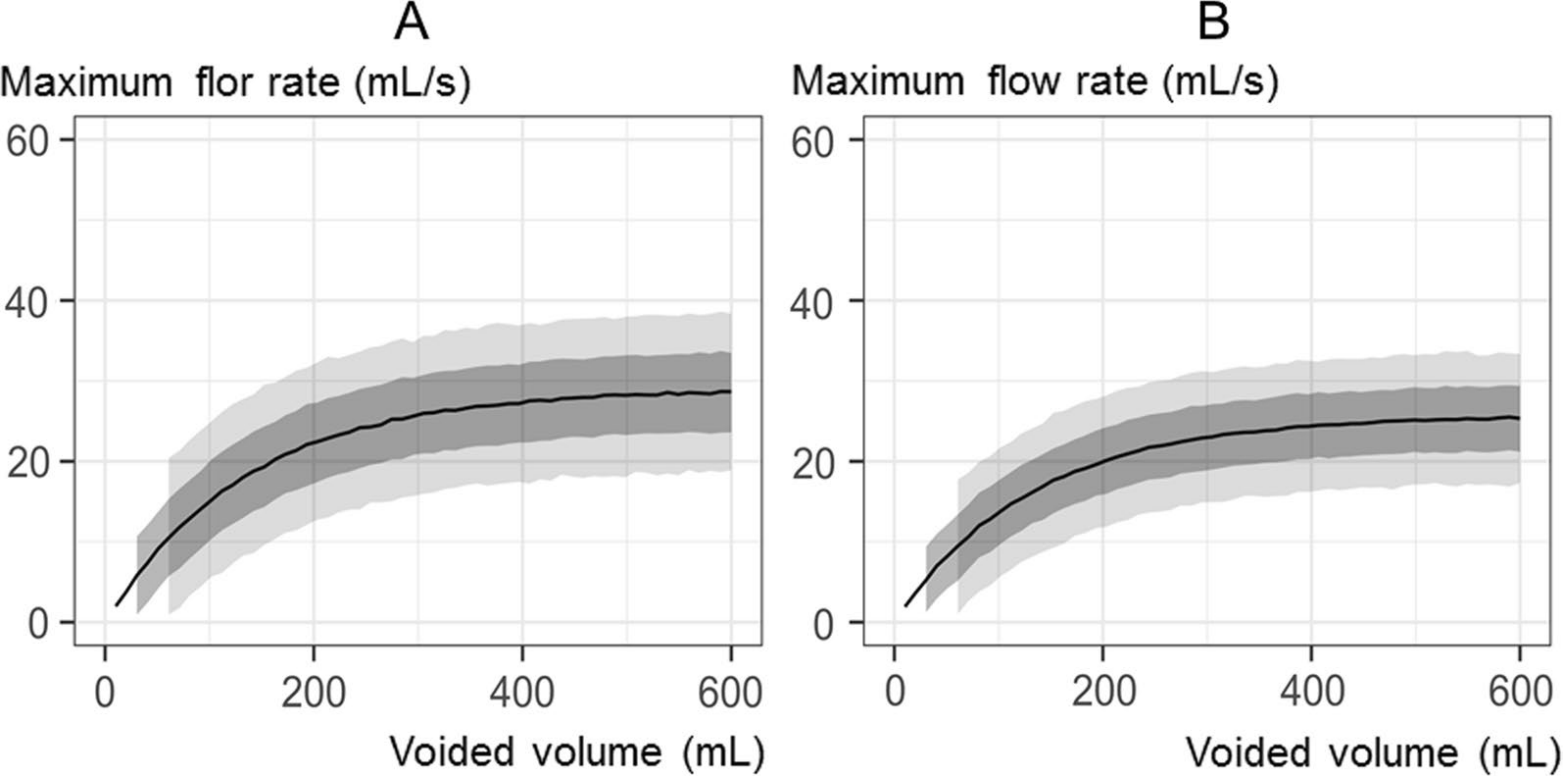
Nomograms on voided volume and maximum flow rate: **A** younger group (20–49 years; mean: 4.92 mL/s) and **B** older group (50–59 years; mean: 4.04 mL/s), with dark gray area representing ± 1 SD and light gray area representing ± 2 SD

In all age groups, the relationship between VV and MFR strongly correlated with the quadratic curve for all participants (Fig. 3A and B). The formula for the younger (20–49 years) group was *Y* = 28.99 {1 − exp(− 0.01 × *X*)} and that for the older group (50– 59 years) was *Y* = 25.67 {1 − exp(− 0.01 × *X*)}.

## Discussion

We successfully developed new nomograms of urinary volume and flow based on the data of a sufficient number of Japanese men without LUTS and multiple flows per participant with clear characteristics. Our nomograms are almost consistent with previous nomograms, with some differences. The 2 previous nomograms were fit for nonlinear regression models, similar to ours [10, 11]. In those nomograms, including ours, the increase in MFR is very low, at approximately ≥ 400 mL. However, the Liverpool nomogram was fit for a linear regression model. MFR gradually increased with VV up to 600 mL [12], which can be attributed to the data of single micturition per participant who was instructed to attend with full bladder. Our nomogram compensates for the shortcomings of previous nomograms and may be the most ideal nomogram to screen men aged < 60 years for voiding dysfunction.

The urinary flow may be different among races and/or ages. Although the mean MFR was around 25 mL/s at approximately 400 mL in the previous Japanese nomogram [11] and ours, the mean MFR was around 30 mL/s at approximately 400 mL in the Canadian [10] and Liverpool nomograms [12]. One of the reasons might be the difference in body mass index, which may affect bladder outlet condition and detrusor contraction. BMI is higher in Western populations than in Asian populations [16]. A higher BMI correponds to a higher prevalence of metabolic syndrome, which may cause peri-urethral fibrosis that contributes to bladder outlet obstruction [17]. If bladder outlet obstruction occurs, the bladder goes through three stages: an initial hypertrophy phase, a subsequent compensation, and a late decompensation [18]. In the initial hypertrophy phase, enhanced detrusor contraction may increase urinary flow. Hence, Western populations may be more likely to be in this state than Japanese populations. In the study of the Liverpool nomogram, the MFRs decreased with age (1.0–1.6 mL/s/10 years) [12]. In previous studies that enrolled relatively younger participants including teenagers, the mean MFRs were relatively higher. In a Thai study, the participants were 18–30 years old, and the mean MFR was 31.2 ± 9.0 mL/s [19]. In an Austrian study, the participants were 18 years old, and the mean MFR was 28.4 ± 9.7 mL/s [20]. In an Indian study, the participants were 15–40 years old, and the mean MFR was 27.3 ± 6.7 mL/s [21]. However, in another Indian study, the participants were 15–50 years old, and the mean MFR was 22.8 ± 9.3 mL/s, which was similar to our study [22]. In the present study, the mean MFR was significantly lower in the 50–59-year age group (~ 22 mL/s) than in the younger groups (~ 24 mL/s), but the mean MFRs were not significantly different among the 20–29-, 30–39-, and 40–49-year age groups. It might be because of the prostate volume. A previous cohort study in Japan demonstrated that the proportion of men older than 50 years with prostate larger than 20 mL was 35%, whereas that of men aged 40–49 years was 20.0% [23].

MFR gradually increased with VV to a certain threshold, followed by a decrease in 66 out of 101 participants (167 out of 894 data sets). In these participants, the MFR decreased by 3.08 ± 2.5 mL/s at more than the threshold VV (~ 300 mL) in comparison with the MFR at the threshold. In the Indian study, the mean MFR was lower at > 750 mL than at ≤ 750 mL [21]. In the Austrian study, the threshold was 550 mL [20]. One of the reasons may be that bladder overdistension leads to weaker detrusor contraction, which was demonstrated in the animal experiment using foxhounds [24].

The present study has some limitations that need to be addressed. First, we did not collect data from women, but we have a plan to study the use of P-Flowdiary® in women, who can utilize the optional portable toilet seat. Second, we did not collect data from men older than 60 years. However, defining “healthy” micturition is not easy because the simple consistency between LUTS and urinary flow may be lower with age [14]. Lower urinary tract dysfunction due to benign prostatic hyperplasia, overactive bladder, and underactive bladder influences consistency in aged men. The “healthy” flow curve of the 50–59-year age group or a treatment target of aged men with lower urinary tract dysfunction may be an ideal. Therefore, the nomograms of men younger than 60 years might be enough.

In conclusion, the quadratic regression model was fit for the nomogram of the relationship between MFR and VV. The nomogram, which can predict MFR by VV in Japanese adult men aged 20–59 years without LUTS, is a useful tool to assist in diagnosing voiding dysfunction.

## Data Availability

The data that support the findings of this study are available from the corresponding author upon reasonable request.

## Abbreviations

LUTS: Lower urinary tract symptoms;
IPSS: International prostate symptom score
OABSS: Overactive bladder symptom score
UFM: Uroflowmetry
FVC: Frequency volume chart
VV: Voided volume
MFR: Maximum flow rate
